# Cationic Polymers Inhibit the Conductance of Lysenin Channels

**DOI:** 10.1155/2013/316758

**Published:** 2013-09-28

**Authors:** Daniel Fologea, Eric Krueger, Steve Rossland, Sheenah Bryant, Wylie Foss, Tyler Clark

**Affiliations:** Boise State University, Department of Physics, 1910 University Drive, Boise, ID 83725-1570, USA

## Abstract

The pore-forming toxin lysenin self-assembles large and stable conductance channels in natural and artificial lipid membranes. The lysenin channels exhibit unique regulation capabilities, which open unexplored possibilities to control the transport of ions and molecules through artificial and natural lipid membranes. Our investigations demonstrate that the positively charged polymers polyethyleneimine and chitosan inhibit the conducting properties of lysenin channels inserted into planar lipid membranes. The preservation of the inhibitory effect following addition of charged polymers on either side of the supporting membrane suggests the presence of multiple binding sites within the channel's structure and a multistep inhibition mechanism that involves binding and trapping. Complete blockage of the binding sites with divalent cations prevents further inhibition in conductance induced by the addition of cationic polymers and supports the hypothesis that the binding sites are identical for both multivalent metal cations and charged polymers. The investigation at the single-channel level has shown distinct complete blockages of each of the inserted channels. These findings reveal key structural characteristics which may provide insight into lysenin's functionality while opening innovative approaches for the development of applications such as transient cell permeabilization and advanced drug delivery systems.

## 1. Introduction

Pore-forming toxins (PFTs) are sophisticated and potent virulence factors evolved in all kingdoms of life as part of the innate defense-offense system [1–6]. PFTs from different subfamilies do not necessarily share sequence or structural homology [7–9], but their common behavior relies on a series of complex events that induce strong disturbances of the permeability function of cell membranes [10–12]. Bacterial and eukaryotic PFTs essentially function as transporters that kill the host cells by simply introducing nonselective transmembrane pores that contribute to the intracellular delivery of toxic compounds or simply dissipate the electrochemical gradients [10–12]. The intensive study of PFTs is motivated by the need to understand their complex mechanisms of action and how to prevent their lethal activity. An equally compelling reason is that the unique transport capabilities of native and reengineered PFTs provide a strong framework for developing biotechnological applications ranging from intended cell permeabilization to single-molecule sensors [13–18]. Early investigations of PFTs have concluded that their applicability as highly specific and efficient tools in biology would be dramatically improved if regulatory mechanisms similar to ion channels were incorporated within their structures [19, 20]. The addition of such features would allow control over the transport through natural or artificial bilayer lipid membranes (BLMs) and would open novel avenues for exploiting applications such as triggering biochemical reactions, developing novel biosensing platforms, or designing advanced systems for drug delivery [13, 16, 17, 19–22]. Regulation by voltage, ligands, or other external conditions is an intrinsic feature of ion channels [23–26], but their use as controlled transporters outside their native environment is limited by their high selectivity, extremely weak capability of macromolecular transport, and difficult reconstitution in artificial systems. In contrast, PFTs are typically larger and less selective than ion channels and maintain prolonged functionality upon facile insertion into artificial BLMs. Although their apparent lack of regulation is a major limitation for controlled transport applications, some remarkable exceptions are noted. Lysenin, a 297-amino-acid PFT extracted from the earthworm *E. foetida *[27–30], constitutes an excellent candidate for controlling the transport through lipid membranes. Lysenin self-assembles to form stable and large conductance pores (~3 nm diameter [6, 30]) into artificial and natural membranes containing sphingomyelin (SM) [28, 31, 32]. The biological function of lysenin has not yet been uncovered, but its cytolytic and hemolytic activities are indicative of a PFT [33]. The channel's properties and behaviors have been recently characterized [31, 34–37], and the investigations revealed considerable similarities with ion channels and functionalities uncommon among PFTs: asymmetrical voltage-induced gating [31, 35], reversible conductance inhibition induced by interactions with multivalent cations [34, 36], a strong dependence of the voltage-induced gating on temperature [37], and an unusual hysteresis of the open-close transition [37]. The exact mechanism of this particular type of conductance modulation by multivalent ions is unknown. It is assumed that primary electrostatic interactions between multivalent ions and one or more binding sites present in the channel's structure trigger a cascade of events that lead to conformational changes and consequent adjustments of the channel conductance [34, 36]. In this respect, we hypothesized that voluminous cationic polyelectrolytes may occlude the conductance pathway by electrostatic interactions. Such investigations on the conductance properties of lysenin will test our understanding of nanoscale electromechanics and explore new functionalities suitable for increasingly complex applications such as temporarily accessing the cytoplasm while maintaining cellular integrity. In this line of inquiries, we investigated the conductance properties of lysenin channels in the presence of highly charged cationic polymers, for example branched polyethyleneimine (PEI) and chitosan. The analysis of the changes in conductance induced by polyelectrolytes on populations and individual lysenin channels suggests a multi-step mechanism of interaction with irreversible changes in conductance involving binding and trapping. In addition, the preservation of the inhibitory effect following the addition of charged polymers on either side of the supporting membrane suggests the presence of multiple binding sites within the channel's structure. 

## 2. Materials and Methods

### 2.1. Chemicals

Stock solutions of 100 mg/mL asolectin (Aso), 25 mg/mL SM, 50 mg/mL cholesterol (Chol), and 100 mg/mL diphytanoyl phosphatidylcholine (DiPhytPC) were prepared by dissolving the dried lipids in n-decane. All lipids were purchased from Sigma-Aldrich and Avanti Polar Lipids. Lysenin (Sigma-Aldrich) was prepared as a 0.3 *μ*M stock solution in 100 mM KCl, 20 mM HEPES, and 50% Glycerol. 10,000 MW branched PEI and medium molecular weight chitosan, along with other chemicals were purchased from various distributors and prepared according to the provider's recommendations. 

### 2.2. BLM Formation, Channel Insertion, and Data Recording

A BLM setup comprised of two 1 mL polytetrafluoroethylene (PTFE) reservoirs filled with buffered electrolyte (130 mM KCl, 20 mM Hepes, and pH 7.2, if not otherwise noted) and separated by a thin PTFE film with a small central hole (~70 *μ*m diameter) was used for electrophysiology measurements. A planar BLM was created by painting the hole with small amounts of a mixture of lipids (Aso : SM : Chol, 1 : 0.5 : 0.25 weight ratios). Neutral BLMs have been produced by replacing Aso with DiPhytPC in the lipid mixture and keeping the same weight ratios. The electrical connections were established via two Ag/AgCl electrodes embedded into the electrolyte solution on each side of the BLM and connected to the headstage of an Axopatch 200B amplifier (Molecular Devices). The data was digitized and recorded through a Digidata 1440A digitizer (Molecular Devices) and further analyzed by using pCLAMP 10.2 (Molecular Devices) and Origin 8.5.1 (OriginLab) software packages. BLM formation and stability were monitored by capacitance and seal resistance measurements after which small amounts of lysenin (~0.3 nM final concentration) were added to the *trans *(grounded) side of the BLM under continuous stirring with a low-noise magnetic stirrer (Dual-Dipole Stirplate, Warner Instruments). Channel insertion was monitored by measuring the ionic currents through the BLM in voltage clamp conditions [31, 32, 34–37] (−60 mV bias potential, 1 kHz lowpass hardware filter). The experiment on single channels comprised an Aso-based BLM to which we added minute amounts of lysenin (~50 pM final concentration) without stirring. After the insertion of a few channels in the BLM, uninserted lysenin was removed by flushing the chamber with 30 mL of fresh buffered electrolyte. A steady-state open current signaled the completion of the insertion process, and the integrity of the inserted channels was assessed by analyzing the voltage-induced gating at positive voltages [35]. All experiments were performed at room temperature. 

## 3. Results and Discussions

### 3.1. Charged Polymers Block the Ionic Transport through Lysenin Channels Inserted into Planar BLMs

**Figure 1:**
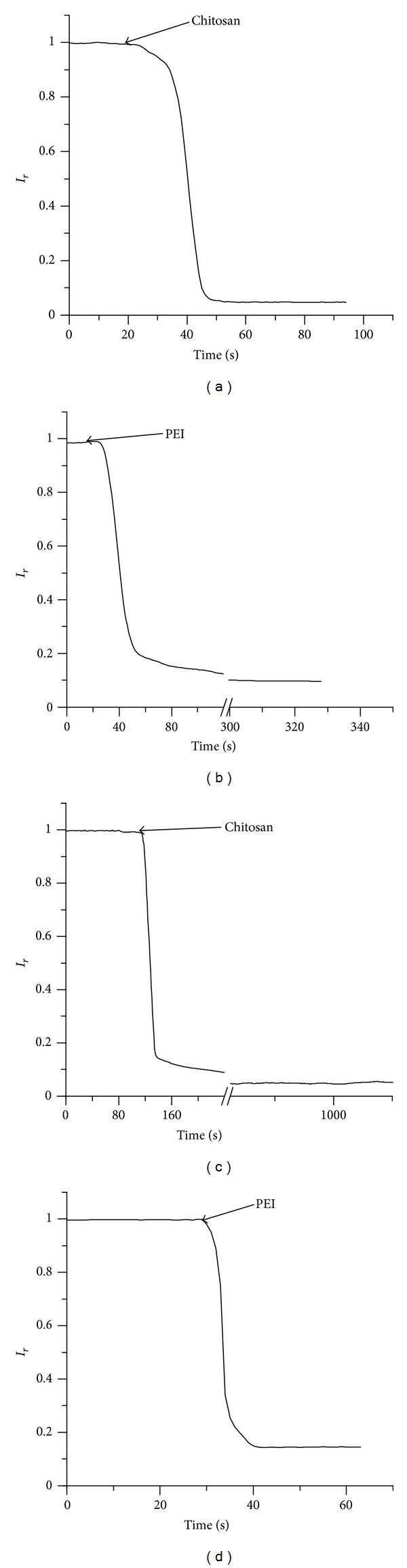
*Trans* addition of chitosan (8 *μ*M final concentration) and PEI (4 *μ*M final concentration) inhibits the macroscopic conductance of lysenin channels inserted into Aso-based ((a) and (b)) and neutral ((c) and (d)) BLMs.

The influence of charged polymers on the macroscopic conductance was assessed by recording the time evolution of the macroscopic current upon addition of chitosan (8 *μ*M final concentration) or PEI (4 *μ*M final concentration) to the *trans* chamber at −60 mV bias potential while stirring. The changes in macroscopic conductance were inferred from the evolution of the macroscopic open current *I*(*t*) and expressed as *I*_*r*_ = *I*(*t*)/*I*_max⁡_, where *I*_max⁡_ is the current measured before adding the inhibitor (*I*_max⁡_ varied between −8 nA and −22 nA at −60 mV bias potential). The addition of chitosan rapidly decreased the macroscopic conductance and yielded a ~95% reduction in the open current (Figure 1(a)), and PEI addition quickly reduced the macroscopic current by ~90% (Figure 1(b)). The experimental data in Figure 1 are representative of typical responses recorded after polymer addition. However, multiple experiments performed in identical conditions revealed large variations of the inhibition rate. This may be explained by accounting for effects originating from limited diffusion and from the inability to precisely control mixing. Nonetheless, the values of *I*_*r*_ recorded at equilibrium yielded a relative standard error less than 7% for each of the four experimental sets. 

The blockage of ionic transport through lysenin channels induced by the addition of PEI and chitosan resembles the inhibitory effects of multivalent ions [34, 36] and poly-L-lysine [38]. Single-channel experiments have shown that multivalent metal cations (such as lanthanides and alkaline earth metals) modulate the lysenin transport properties by promoting conformational changes that switch the channels from conducting (open) to subconducting (partially closed) or nonconducting (closed) states, but the removal of the inhibitors by flushing, chelation, or precipitation quickly restored the conducting properties [34, 36]. In contrast, the induced-inhibition with chitosan or PEI was not found to be reversible by buffer exchange, and the open currents (at −60 mV bias voltage) maintained the same extremely low level for hours suggesting that the unblocking process was either extremely slow or did not occur at all. This apparent lack of reversibility may be a resultant of the positively charged polymers permanently occluding the channel or interfering with their structural integrity by interacting either with the protein channels or with other structural components of the lipid membrane. Both cationic polymers have the ability to interact with lipid membranes and to disturb their permeability [39–42], which makes them useful as transfecting reagents [43, 44]. Charge interactions play a key role in establishing the initial contact between charged polymers and lipid membranes [39–42], which complicates the analysis since the voltage-induced gating of lysenin is strongly dependent on the lipid environment [31, 36]. The asymmetrical voltage-induced gating that occurs at relatively low positive voltages applied to lysenin channels has been well established [31, 35] as well as the almost complete suppression of voltage gating when neutral lipids are used to produce the supporting BLMs [31, 36]. The lysenin voltage-induced gating is strongly dependent on the BLM composition and is presumed to be conditioned by electrostatic interactions between channels and anionic lipids composing the bilayer [31, 36]. Consequently, the channels might undergo conformational changes owing to interactions between lipids and charged polymers, or surface accumulation of charged polymers may physically occlude the channels and yield the observed inhibition in the macroscopic currents. To investigate the potential implications of such electrostatic interactions between lipids and cationic polymers, we repeated the inhibition experiments with the same electrical conditions but replaced Aso with the neutral DiPhytPC in the BLM. The addition of chitosan (Figure 1(c)) or PEI (Figure 1(d)) to the *trans* chamber once again yielded a strong decrease in the macroscopic currents. These findings suggest that the inhibition of the ionic current is triggered by interactions between charged polymers and lysenin channels as opposed to electrostatic interactions with lipids. In addition, we may conclude that the voltage-induced gating and the inhibition of the ionic current induced by charged polymers are independent processes, as previously demonstrated for multivalent ions [31, 34, 36]. 

Earlier investigations demonstrated that *β*-lactoglobulin (BLG) is removed from lipophilic environments by chitosan [45]. However, those experiments were conducted on lipid monolayers, and it is difficult to presume that chitosan can similarly remove stable lysenin channels from the supporting BLM. In addition, BLG removal from lipid monolayers by chitosan was found to require the presence of anionic lipids to electrostatically interact with the charged chitosan [45]. The identical observed inhibitory effects from charged polymers in the presence of anionic and neutral lipid bilayers suggest that the decrease in the macroscopic conductance of lysenin channels relies on a mechanism that does not involve the removal of channels from the BLM and that electrostatic interactions between the cationic polymers and the lipid membrane are not prerequisites for the inhibition in conductance.

The similarities between chitosan and PEI, in terms of their physical properties, have been analyzed to further understand their interaction with lysenin channels. Both polymers are positively charged at neutral pH, and since they are quite voluminous [46–48], their large size suggests that they may have the ability to simply occlude the lysenin channel. However, the physical occlusion of the channel pathway, in principle, should be a reversible process, and such a blocking mechanism is not supported by the apparent lack of reversibility. Previous work with spermidine and spermine suggests that electrostatic interactions between these highly charged organic cations and lysenin channels induce conformational transitions manifested as subconducting states [34, 36] while maintaining the permeability for the organic cations. Apparently, charge density plays an important role in establishing an inhibitory mechanism based on either full or partial closing of the channels [34, 36]. The polymers used in this study have a charge density lower than Ca^2+^ or Mg^2+^, therefore, an inhibitory mechanism based on channels attaining a stable subconducting state would be expected. The strong inhibition observed at very low concentrations makes the cationic polymers even more efficient blockers than many trivalent metal ions [34, 36] and is not supportive of a blockage mechanism based on channels attaining stable subconducting states. To reconcile these discrepancies, we propose a multistep blocking mechanism of lysenin channels based on electrostatic interactions and physical occlusion. When the positively charged polymer interacts electrostatically with a binding site present within the channel's structure, the strong interaction induces conformational changes that constrict the pore lumen (subconducting or non-conducting state) and traps the polymer in the channel. This electrostatic-induced physical blockage impedes the free ionic flow and manifests as a decrease in macroscopic conductance and open current. The strong electromechanical interactions prevent further release of the trapped polymer and channel reactivation, rendering the process quasiirreversible. 

### 3.2. Charged Polymers and Lysenin Orientation: Evidence of Multiple Binding Sites

**Figure 2:**
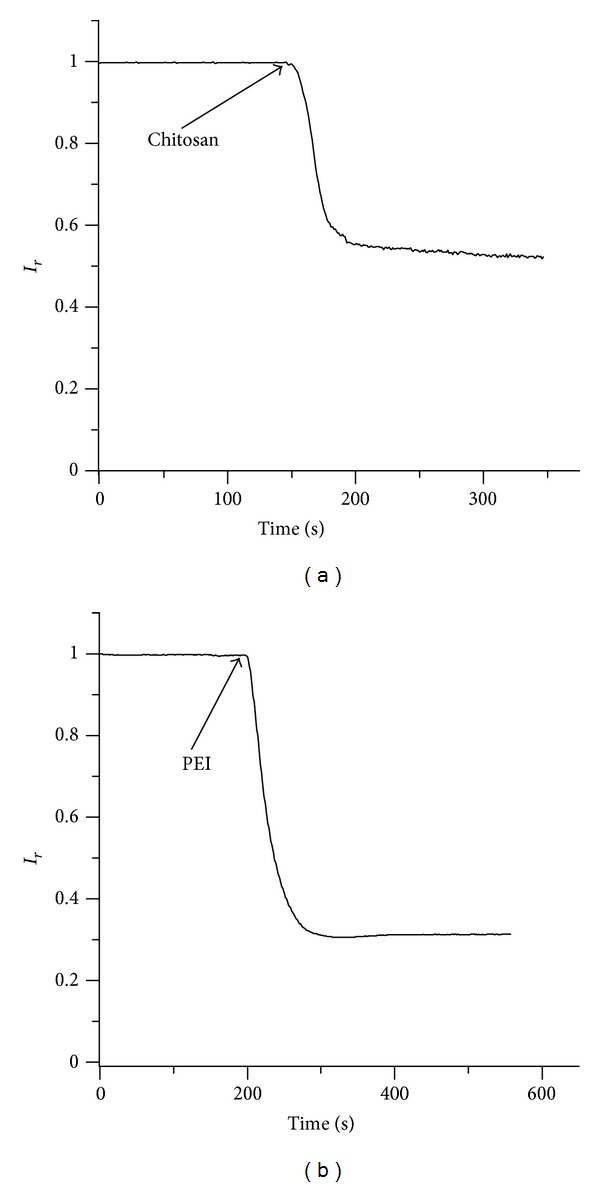
The evolution of the relative macroscopic current through a population of lysenin channels inserted into an Aso-based BLM in the presence of (a) 8 *μ*M chitosan or (b) 4 *μ*M PEI added to the *cis* chamber.

The orientation of the lysenin channels and the direction of the electric field were chosen to facilitate interactions between the channels and the positively charged polymers. The hydrodynamic diameter of the polymers is assumed to be larger than the channel opening, so the results of the investigations thus far suggest that the binding site is positioned closer to the *trans* side. Therefore, an electric field oriented in the opposite direction should discourage interactions between the polymer molecules and the binding site and prevent channel blockage. To examine this reasoning, the polymers were added to the *cis* side while the bias voltage remained −60 mV. Unexpectedly, both chitosan (Figure 2(a)) and PEI (Figure 2(b)) elicited current blockages in lysenin channels inserted into Aso-based BLMs. However, compared to *trans* side additions (as depicted in Figure 1), the macroscopic currents were less severely inhibited (~50% by chitosan, and ~70% by PEI).

This experiment provided supplementary evidence for the channels not being pulled out from the supportive BLM by the cationic polymers. Given the particular structure of the lysenin monomer interacting with SM in a BLM [49], the asymmetric shape, and the hydrophilic C-terminus, it is unlikely that either of the charged polymers could successfully pull the lysenin channels in either direction through the BLM. Nonetheless, this experiment led us to conclude that the hypothesis of a unique binding site closer to the *trans* side may be no longer valid. Even if we consider that the stretched polymers may accommodate the narrow opening of the lysenin pore, it is doubtful that they would be able to overcome the energy barrier from the electric field inside the channel and traverse the pore. The electric field outside the pore is much smaller, so thermal agitation may initiate interactions, but this mechanism implies a second binding site positioned proximal to the *cis *side of the channel. The result of an opposing electric field and thermal agitation creates a lower localized concentration of polymer near the BLM yielding the weaker inhibition observed for *cis. *By intermittently reversing or removing the bias voltage, we observed a further reduction in the current, reaching similar levels as those of the *trans* addition. 

**Figure 3:**
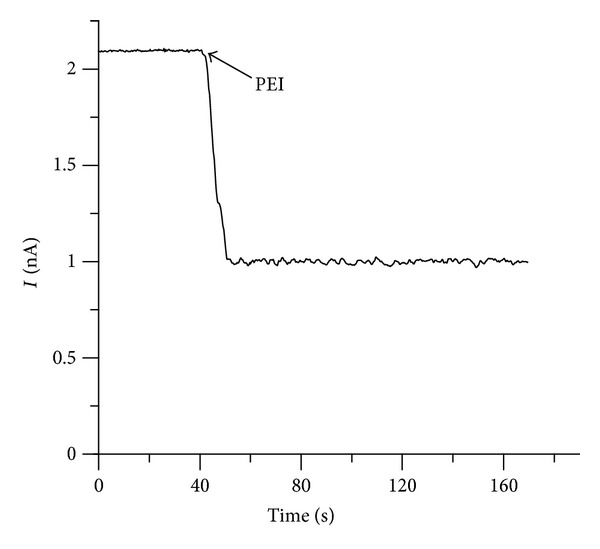
Addition of PEI (10 *μ*M final concentration) to the *cis* side inhibits the macroscopic current through lysenin channels inserted in a neutral BLM biased by 30 mV.

The inhibition observed upon adding the polymers to the *trans* side of a negatively biased BLM supports the hypothesis of a binding site situated close to the *trans* opening of the channel only if the polymers are unable to thread the pore. A binding site situated near the *cis* side of the channel would be accessible to polymers added to the *trans* side if they were completely straightened by an appropriately directed electric field so that they may negotiate the narrow pore and interact with the distal binding site. However, an opposite electric field should prevent the polymer's translocation due to the large energy barrier. To examine this possibility, we investigated the interaction of PEI with lysenin channels inserted in a neutral BLM biased by 30 mV. The addition of PEI (10 *μ*M final concentration) to the *trans* side revealed that the conductance was still inhibited (Figure 3) suggesting the existence of a binding site accessible from the *trans* side of the channel without involving threading.

**Figure 4:**
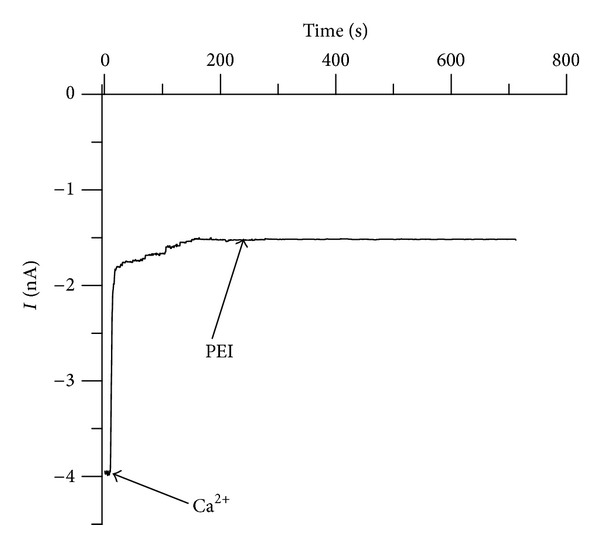
PEI and multivalent ions compete for the binding sites present within the channel structure. PEI (10 *μ*M final concentration) lack the inhibitory capabilities when the binding sites of lysenin have been previously blocked by the addition of Ca^2+^ (40 mM final concentration).

The inhibition of the macroscopic current by either multivalent cations or cationic polymers requires primary electrostatic interactions with the binding sites. After binding, multivalent ions trigger a gate to either fully or partially close the conducting pathway, while the charged polymers are most likely trapped within the channel. In order to determine if multivalent cations and cationic polymers interact with the same binding site to elicit current blockage, we observed the successive interaction of the ligands with lysenin channels inserted into an Aso-based BLM biased by −60 mV. Addition of 40 mM Ca^2+^ to both sides of the BLM decreased the macroscopic current (Figure 4) and forced all the channels to adopt a subconducting state [34]. Addition of PEI (10 *μ*M final concentration) to both sides did not induce a supplementary decrease of the open current (Figure 4), indicating an absence of further conductance inhibition. The most reasonable explanation for this behavior is that excess Ca^2+^ in solution is occupying all the available binding sites thus inhibiting further interaction with PEI. We cannot exclude the possibility that the subconducting lysenin channels cannot undergo further conformational changes and consequent full closing even if they bind PEI. However, such interaction with the voluminous polymers would imply a decrease of the open current, which was not observed. 

### 3.3. Individual Blockage of Conductance: Single-Channel Analysis

**Figure 5:**
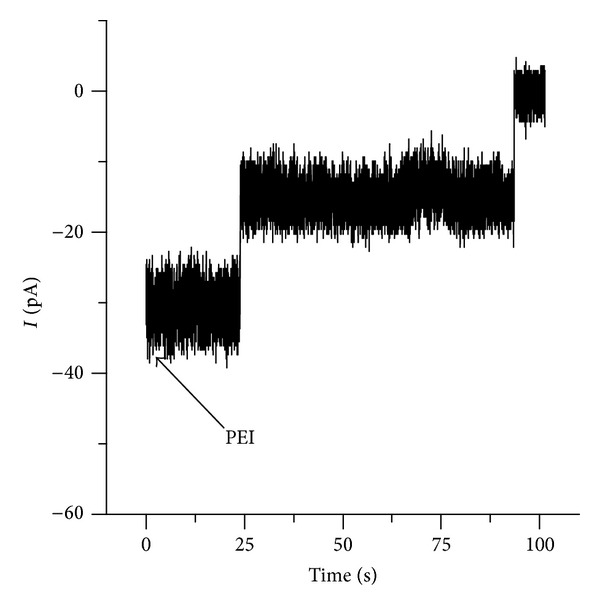
Addition of PEI (10 *μ*M final concentration) to the *trans* side of an Aso-based BLM containing two lysenin channels induces a step-wise and unitary decrease in the open current and indicates quick and individual interactions of PEI with each of the two inserted channels.

Previous experiments with single lysenin channels have shown that trivalent metals eliminate the conductance of individual channels in a single step, while divalent metals induce conformational changes leading to stable subconducting states [34]. In addition, highly charged organic ions were found to provoke subconducting transitions similar to the effects from divalent ions [34, 36]. Presumably, the inhibitory effect of multivalent cations is based on ligand-induced gating that is, the multivalent ions interact with one or more binding sites and trigger the movement of a gate such that the channel is partially or fully closed [34, 36]. The interaction with charged polymers presented here is different although apparently involves the same binding sites. With few exceptions, maximum inhibition of the microscopic currents by divalent metals or organic ions requires bulk concentrations in the mM range [34, 36]. In addition, the inhibition process proved to be reversible, and the conductance properties have been rapidly restored following the removal of the inhibitory ions from the bulk [34, 36]. We hypothesized that the interaction between the charged polymers and lysenin channels implies binding and trapping, which is consistent with the apparent irreversibility. However, we have not yet identified if the charged polymers modify the individual channel conductance continuously or in one or more discrete steps. Therefore, we explored the interaction between PEI and individual lysenin channels. The insertion of two lysenin channels in an Aso-based BLM bathed by 50 mM NaCl was performed as described in the previous sections. *Trans* addition of PEI (10 *μ*M final concentration, no stirring) revealed that the ionic current (recorded at −60 mV bias potential and 1 ms^−1^ sampling rate) vanished by undergoing a step-wise transition (Figure 5), consistent with distinct complete blockages of each of the inserted channels. The discrete transformation of the ionic current qualitatively resembles the interaction with trivalent metal ions [34]. However, it is possible that each step consists of multiple steps unresolved at the sampling rate used. 

## 4. Conclusions

Positively charged polymers were found to inhibit the intrinsic transport capabilities of lysenin channels in an irreversible manner and reinstate the intrinsic barrier function of the supporting BLM. Our results support a blocking mechanism based on electrostatic interactions between charged polymers and multiple binding sites located in the proximity of the channel mouth, followed by conformational changes that lead to trapping. However, we cannot completely rule out a mechanism triggered by interactions between charged polymers and lipids in the membrane, which may locally change the electrical and mechanical properties therefore affecting the lysenin channel functionality. Further work in this direction will establish the validity of the binding-trapping hypothesis advanced here. Nonetheless, this work provides supplementary information about the channel structure and functionality, which may prove to be helpful in deciphering the unknown physiological role of lysenin. The mechanistic model of interaction proposed here can be extended for understanding the interaction between ion channels or porins with charged polymers. In addition, this work demonstrates that lysenin channels constitute an excellent experimental model for nanoscale control over the transport of ions and biologically significant molecules through BLMs.
